# Dysregulated palmitic acid metabolism promotes the formation of renal calcium-oxalate stones through ferroptosis induced by polyunsaturated fatty acids/phosphatidic acid

**DOI:** 10.1007/s00018-024-05145-y

**Published:** 2024-02-12

**Authors:** Rui Wang, Jingdong Zhang, Haotian Ren, Shiyong Qi, Linguo Xie, Haijie Xie, Zhiqun Shang, Chunyu Liu

**Affiliations:** 1 Department of Urology, Tianjin Institute of Urology, The Second Hospital of Tianjin Medical University, Tianjin, China; 2 Department of Physiology and Pathophysiology, Tianjin Medical University, Tianjin, China

**Keywords:** Renal calcium oxalate stone, Palmitic acid, Ferroptosis, Metabolism, Multiomics

## Abstract

The pathogenesis of renal calcium-oxalate (CaOx) stones is complex and influenced by various metabolic factors. In parallel, palmitic acid (PA) has been identified as an upregulated lipid metabolite in the urine and serum of patients with renal CaOx stones via untargeted metabolomics. Thus, this study aimed to mechanistically assess whether PA is involved in stone formation. Lipidomics analysis of PA-treated renal tubular epithelial cells compared with the control samples revealed that α-linoleic acid and α-linolenic acid were desaturated and elongated, resulting in the formation of downstream polyunsaturated fatty acids (PUFAs). In correlation, the levels of fatty acid desaturase 1 and 2 (FADS1 and FADS2) and peroxisome proliferator-activated receptor α (PPARα) in these cells treated with PA were increased relative to the control levels, suggesting that PA-induced upregulation of PPARα, which in turn upregulated these two enzymes, forming the observed PUFAs. Lipid peroxidation occurred in these downstream PUFAs under oxidative stress and Fenton Reaction. Furthermore, transcriptomics analysis revealed significant changes in the expression levels of ferroptosis-related genes in PA-treated renal tubular epithelial cells, induced by PUFA peroxides. In addition, phosphatidyl ethanolamine binding protein 1 (PEBP1) formed a complex with 15-lipoxygenase (15-LO) to exacerbate PUFA peroxidation under protein kinase C ζ (PKC ζ) phosphorylation, and PKC ζ was activated by phosphatidic acid derived from PA. In conclusion, this study found that the formation of renal CaOx stones is promoted by ferroptosis of renal tubular epithelial cells resulting from PA-induced dysregulation of PUFA and phosphatidic acid metabolism, and PA can promote the renal adhesion and deposition of CaOx crystals by injuring renal tubular epithelial cells, consequently upregulating adhesion molecules. Accordingly, this study provides a new theoretical basis for understanding the correlation between fatty acid metabolism and the formation of renal CaOx stones, offering potential targets for clinical applications.

**Supplementary Information:**

The online version contains supplementary material available at 10.1007/s00018-024-05145-y.

## Introduction

Urolithiasis is one of the most common urological diseases, and its incidence has exhibited rapid growth in recent years. Annual new incidence of kidney stones is approximately 100–400/10^5^ in Europe, 1-19.1% in Asia, and 5.8% in China [1–3]. In addition, the substantial recurrence rates of urolithiasis ranging from 6 to 23% within the first year post-treatment and surging to 50% after a five-year interval, present a significant treatment challenge [4, 5]. Urolithiasis can be categorized into two principal types-calcium stones and non-calcium stones, with calcium oxalate (CaOx) stones constituting the majority and accounting for 65–80% of cases [5, 6].

The etiology and pathogenesis of renal CaOx stones are complex and unclear, and many factors are involved, including metabolic, genetic, environmental factors (such as the geographical region, climate, occupation, and diet), and local factors in the urinary tract (such as anatomical abnormalities, infection, obstruction, and foreign bodies). At present, effective and accurate preventive and therapeutic strategies for CaOx stones are still lacking.

Recent reports suggest that urolithiasis, as a systemic disorder, is closely related to metabolic syndrome (MS) [7, 8]. MS is associated with various metabolic disorders, including obesity (mainly abdominal obesity), fasting and postprandial hyperglycemia, hypertension, and dyslipidemia. In our previous report, the proportion of patients diagnosed with MS among urolithiasis patients was estimated at 29.9% (272/910) [9].

Studies have revealed that dyslipidemia is an independent risk factor for the formation of urinary calculi. In a retrospective study involving 52,184 patients, abnormal levels of blood lipids were found to correlate with an increased risk of kidney stones (hazard ratio, 2.2; 95% confidence interval,1.9–2.5; *p* < 0.001) [10]. Additionally, in our previous study, the proportion of patients accompanied by dyslipidemia among urolithiasis patients was estimated at 61.8% (562/910) [9]. This rate is much higher than the overall incidence of dyslipidemia in Chinese adults (≥ 35 years old), which stands at 34.7%. Although the correlation between dyslipidemia and the incidence of urolithiasis has become evident, the precise mechanism whereby dyslipidemia influences stone formation remains unclear.

Free fatty acids (FFAs), which encompass saturated fatty acids (SFAs), monounsaturated fatty acids (MUFAs), and ω 6- and ω 3-polyunsaturated fatty acids (PUFAs), are intermediate factors in lipid metabolism. Pathological upregulation of serum FFAs can stimulate oxidative stress, induce excessive cellular release of reactive oxygen species (ROS), and damage renal tubular cells [11, 12]. Mitochondrial flavoprotein long-chain acyl-CoA dehydrogenase can catalyze the mitochondrial FA oxidation and directly produce H_2_O_2_ in diseased kidneys [13]. Injury to renal tubular epithelial cells is considered an important cause of renal stone formation. Such injuries cause changes in the membrane structure and adhesion properties of these cells, thereby promoting the deposition of stone crystals [14].

Previously, to reveal the metabolic differences between patients with renal CaOx stones and healthy people, nontargeted metabolomics has been performed on urine samples from these individuals. Consequently, palmitic acid (PA), which is a major SFA, has been found significantly increased in the urine of these patients [15]. However, whether PA is involved in the formation of renal CaOx stones is unclear. Thus, this study aimed to mechanistically assess for such an involvement.

## Methods and materials

### Ethical statement

This study was pre-approved by the Ethics Committee of the Second Hospital of Tianjin Medical University (No. KY2022K060). All participants were informed of the study and signed the study consent form for the collection and analysis of their urine, serum, and stone samples.

### Human urine and serum samples

Urine samples were collected from 137 patients with renal CaOx stones and 103 healthy controls. In our previous study, we conducted untargeted metabolomics on these urine samples and presented the discovery of PA as a notably differential metabolite in patients with renal CaOx stones [15]. Among these 137 patients, 82 and 55 patients were new-onset and relapse cases, respectively. The clinical characteristics of these patients and healthy controls are presented in Table S1.

Additionally, in this study, serum samples were obtained from 33 new-onset patients, 32 relapsed patients, and 20 healthy controls to assess whether serum PA level was also higher in patients with renal CaOx stones than in healthy individuals. These serum samples were collected after an overnight fast and stored at -80 ℃. The baseline features of each group are summarized in Table S2. Patients diagnosed with secondary causes of urolithiasis, monogenic-induced nephrolithiasis, renal dysfunction, urinary tract infection, liver disease, parathyroid dysfunction, anemia, cancer, congenital renal or urinary tract anomalies, or pregnancy were excluded from the study.

### Animal experiments

Animal experiments were conducted following the approval and protocol of the Second Hospital of Tianjin Medical University and the Declaration of Helsinki. Wild-type male mice, aged 8 weeks and weighing 22–24 g, were obtained from SPF Biotechnology (SCXK 2019-0010), and then randomly assigned to the PA-treated and untreated group. To induce renal injury by using PA, mice were placed on a regular diet containing 7.5% PA (H8780, Solarbio) by weight for 20 weeks. The diet composition was formulated based on our preliminary experiments and previous reports [16–18]. The control group was fed a regular diet. After the 20-week period, all the mice were sacrificed, and blood samples (> 0.5 mL each) were collected through cardiac puncture and put into 1.5 ml tubes with heparin. Additionally, their kidneys were harvested.

Via renal histological analysis and continuous monitoring of renal function, the above-mentioned regimen was verified to induce injury to renal tubular epithelial cells of these mice fed with PA. Then, mice in each group were subjected to daily intraperitoneal injections of glyoxylic acid (Gly) (G10601, Sigma, 80 mg/kg dissolved in sterile saline) to establish the model of renal CaOx stones [19, 20].

Within the 20 weeks of feeding with PA, ferrostatin-1 (Fer-1) (HY 100,579, MCE, 2 mg/kg dissolved in 5% DMSO and then diluted in sterile saline), ζ-Stat (HY-123,979, MCE, 1 mg/kg dissolved in sterile saline) and GW6471 (HY-114,263, MCE, 10 mg/kg dissolved in 5% DMSO diluted in sterile saline) were administered daily via intraperitoneal injections starting from the 16th week until the establishment of the renal CaOx stone model. The corresponding control groups received an equivalent volume of sterile saline or DMSO (< 5%).

### Cell experiments

HK-2 human renal tubular epithelial cells were purchased from the American Type Culture Collection and maintained in DMEM/F-12 containing 10% fetal bovine serum (Gibco) at 37 ℃ with 5% CO_2_. To induce cell injury, they were plated in 96-well plates and then treated with PA (P0500, Sigma) (IC50 estimated at approximately 400 µM) for 24 h. Afterward, their viability was assessed using the MTT assay (0.5 mg/ml) (M5655, Sigma) according to the instructions of the manufacturer. For cell experiments, such as the extraction of mRNA or proteins, HK-2 cells were seeded at a density of 2 × 10^5^ cells/well in a 6-well plate.

### Immunohistochemistry

Formalin-fixed paraffin-embedded sections of 5 μm thickness were used. After deparaffinization, rehydration, and heat-mediated antigen retrieval by using 10 mM sodium citrate, endogenous peroxidase activity was blocked using 3% hydrogen peroxide. Then the sections were incubated overnight at 4 °C with appropriately diluted primary antibodies, followed by incubation with a horseradish peroxidase (HRP)-conjugated secondary antibody (PV-6000, Zsbio). A DAB kit (ZLI-9018, Zsbio) was used for visualization, following the instructions of the manufacturer. The antibodies used are presented in Table S3.

### Immunocytofluorescence

HK-2 cells were seeded into 12-well glass plates at a density of 30,000/well. After they were fixed at room temperature for 30 min by using 4% paraformaldehyde and then permeabilized using 0.1% Triton-100 for 15 min. Afterward, the cells were subjected to immunofluorescence staining. For dual renal immunofluorescence staining for phosphatidyl ethanolamine binding protein 1 (PEBP1) and 15-lipoxygenase (15-LO), kidney sections were subjected to antigen retrieval and then incubated overnight at 4 ℃ with primary antibodies against these proteins, followed by incubation with goat anti-mouse and anti-rabbit secondary antibodies (Table S3).

### Hematoxylin and eosin (HE), periodic acid-Schiff (PAS), and Von Kossa staining

Kidney sections were subjected to HE staining to assess the injury to the renal tubular epithelial cells. To observe the structural changes in the epithelium and brush border of renal tubules, a PAS assay kit (G1280, Solarbio) was used according to the instructions of the manufacturer. For quantitative analysis of CaOx crystal deposition, a Von Kossa kit (150,687, Abcam) was used according to the instructions.

### Monitoring of serum creatinine (scr) and blood urea nitrogen (BUN) levels of mice

To monitor the changes in the renal function of mice fed with PA, Scr and BUN assay kits (AS0174 and AS0172, SAB) were used according to the instructions of the manufacturers. For these assessments, blood was collected from mice at 5, 10, 15, and 20 weeks.

### Enzyme-linked immunosorbent assay (ELISA)

ELISA kits were adopted to measure the levels of PA (69-26485, Mskbio), hyaluronic acid (HA) (69-22935, Mskbio), phosphatidic acid (69-99778, Mskbio), and arachidonic acid (AA) (69-10011, Mskbio) following the instructions.

### RNA-sequencing

To examine the effect of PA on gene transcription, three paired and biologically duplicated HK-2 cell samples that were PA-treated or untreated were collected. The total RNA of each sample was extracted with TRIzol (15,596,026, Invitrogen), and then subjected to RNA high-throughput sequencing by Cloud-Seq Biotech. Briefly, RNA libraries were constructed and library sequencing was then performed on an Illumina Novaseq 6000 instrument by using 150 cycles. High-quality clean reads were aligned to the reference genome (UCSC HG19). Subsequently, guided by the Ensembl GTF-gene annotation file, the Cuffdiff software (a component of Cufflinks) was used to derive gene-level FPKM values, representing the mRNA levels. Differential mRNA levels were identified based on fold change (FC ≥ 2) and a significance threshold of *p* < 0.05. Accordingly, 797 and 469 genes were upregulated and downregulated in PA-treated HK-2 cells, respectively. The dataset was deposited in the Gene Expression Omnibus under accession number GSE240279, as per NCBI guidelines.

### Quantitative reverse transcription–polymerase chain reaction (qRT-PCR)

Total RNA was extracted from cells by using the TRIzol reagent (15,596,026, Invitrogen), and then 5 µg of total RNA was reverse-transcribed in a 20 µL volume by using oligo dT primers and the Revertaid First-strand cDNA synthesis kit (K1622, ThermoFisher). The resulting cDNA was then subjected to PCR analysis by using the Applied Biosystems 7900 Real-Time PCR System (ThermoFisher) and the Faststart Universal SYBR green master mix (4,913,914,001, Roche). The relative expression levels of target genes were calculated using the 2-ΔΔ^Ct^ method. GAPDH was used as an internal control. The sequences of the primers used are shown in Table S4.

### Western blotting

HK-2 cells that were PA-treated or untreated were lysed using RIPA buffer (R0010, Solarbio) containing 1% cocktail proteinase inhibitors (78,441, ThermoFisher). Then, samples with equal amounts of protein (30 µg) were resolved using SDS-polyacrylamide gel electrophoresis (A1010, Solarbio) and transferred onto PVDF membranes (IPVH00010, Millipore). After blocking the membranes with 5% bovine serum albumin (BSA) (A8020, Solarbio), they were incubated with primary antibodies at 4 °C overnight. Afterward, the membranes were washed three times with Tris-buffered saline containing 0.05% Tween and then incubated with HRP-conjugated secondary antibodies (1:10,000 dilution) at room temperature for 1 h. Target bands were visualized using an ECL system (5200, Tanon). The expression levels of the target proteins were calculated using Image J based on the gray values of the corresponding bands. All the primary antibodies used are listed in Table S3.

### Assessment of cellular ROS, Fe^2+^, lipid ROS, glutathione (GSH), and 4-hydroxynonenal (4-HNE) levels, and the activity of glutathione peroxidase 4 (GPX4)

Cellular ROS levels were assessed using the 2′,7′-dichlorofluorescin diacetate probe (D6470, Solarbio). Cellular Fe^2+^ levels were measured using an iron assay kit (ab83366, Abcam) by following the instructions of the manufacturer. In this assay, Fe^2+^ reacts with an iron probe, forming a stable colored complex with a maximum absorption peak at 593 nm. Cellular glutathione levels were assessed using a GSH/GSSG ratio detection assay kit (ab138881, Abcam). This assay employs a proprietary non-fluorescent dye, which undergoes significant fluorescence upon reacting with GSH, with the absorbance measurements performed at 490/520 nm.

Cellular 4-HNE levels were measured using a lipid-peroxidation (4-HNE) assay kit (ab238538, Abcam). This kit measures the 4-HNE adducts in cell lysates by comparing the absorbance of the samples to a known 4-HNE-BSA standard curve. The GPX4 activity was evaluated using an HT glutathione peroxidase assay kit GPX4 activity (7512-100-K, Trevigen). To evaluate intracellular and membrane lipid ROS accumulation, cells were stained with BODIPY (581/591) c11 (D3861, Invitrogen). All the measurements were performed by following the instructions.

### Co-immunoprecipitation

Cells treated as indicated were collected and then lysed using 500 µL cell lysis buffer (88,804, ThermoFisher). Subsequently, 2–10 ug (per 500–1000 ug protein) of mouse anti-PEBP1 primary antibody (101,504, Santa Cruz) was added to the samples, followed by overnight incubation at 4 ℃. Next, 25 µL of pre-washed Protein A/G Magnetic Beads were introduced into the samples, which were then incubated at room temperature for 1 h. Afterward, the magnetic beads were isolated and washed. Then, 100 µL of elution buffer was added to the beads, and the mixture was shaken at room temperature for 10 min. Finally, the supernatants were collected to be analyzed via western blotting.

### Transmission electron microscopy

HK-2 cells treated as indicated were fixed at 4 ℃ for 2 h by using 2.5% glutaraldehyde. After the dehydration, permeabilization, embedding, and sectioning, mounting steps, the samples were analyzed by using a transmission electron microscope (SU8100, Hitachi).

### Lipidomics analysis

The medium- and long-chain FA (M/LCFA) profiles of PA-treated HK-2 cells were characterized via lipidomics analysis. To this end, 1 mL of a chloroform-methanol solution was added to the cell samples, followed by sonication for 30 min. Subsequently, 2 mL of a 1% sulfuric acid-methanol solution was introduced to the supernatant, which was then heated at 80 ℃ for 30 min. Afterward, 1 mL of n-hexane was added to extract the total lipid content of the sample. Then, 500 µL of the supernatant was combined with 25 µL of methyl n-nineteenth acid, serving as an internal standard. In the gas chromatography–mass spectrometry (GC-MS) analysis, an injection volume of 1 µL and a split ratio of 10:1 were used. The lipids were resolved using an Agilent DB-WAX capillary column (30 m×0.25 mm ID×0.25 μm) in a GC system. The initial temperature was set at 50 ℃ for 3 min, followed by an increase to 220 ℃ for 5 min at a rate of 10 ℃/min. Helium served as the carrier gas at a flow rate of 1 mL/min.

For quality control, each quality-control sample was randomly inserted to assess the stability of the mass-spectrometry separation and the reliability of the results. Mass spectrometry was performed using an Agilent 7890/5975c gas-mass spectrometer, with the sample-port, ion-source, and transmission-line temperatures set at 280, 230, and 250 °C, respectively, and the electron energy set as 70 eV. Peak areas and retention times were extracted using the MSD ChemStation software, and calibration curves were generated to calculate the concentrations of the M/LCFAs in the samples.

### Statistical analysis

SPSS 21.0 and GraphPad Prism 8.0 were used for data analysis and graph generation. Comparative analyses between groups were executed using either *t*-tests or ANOVA. A *p*-value < 0.05, based on a two-sided test, was considered to indicate statistically significant. All the experiments were independently repeated three times, yielding consistent results.

## Results

### PA was significantly upregulated in patients with renal CaOx stones

**Fig. 1:**
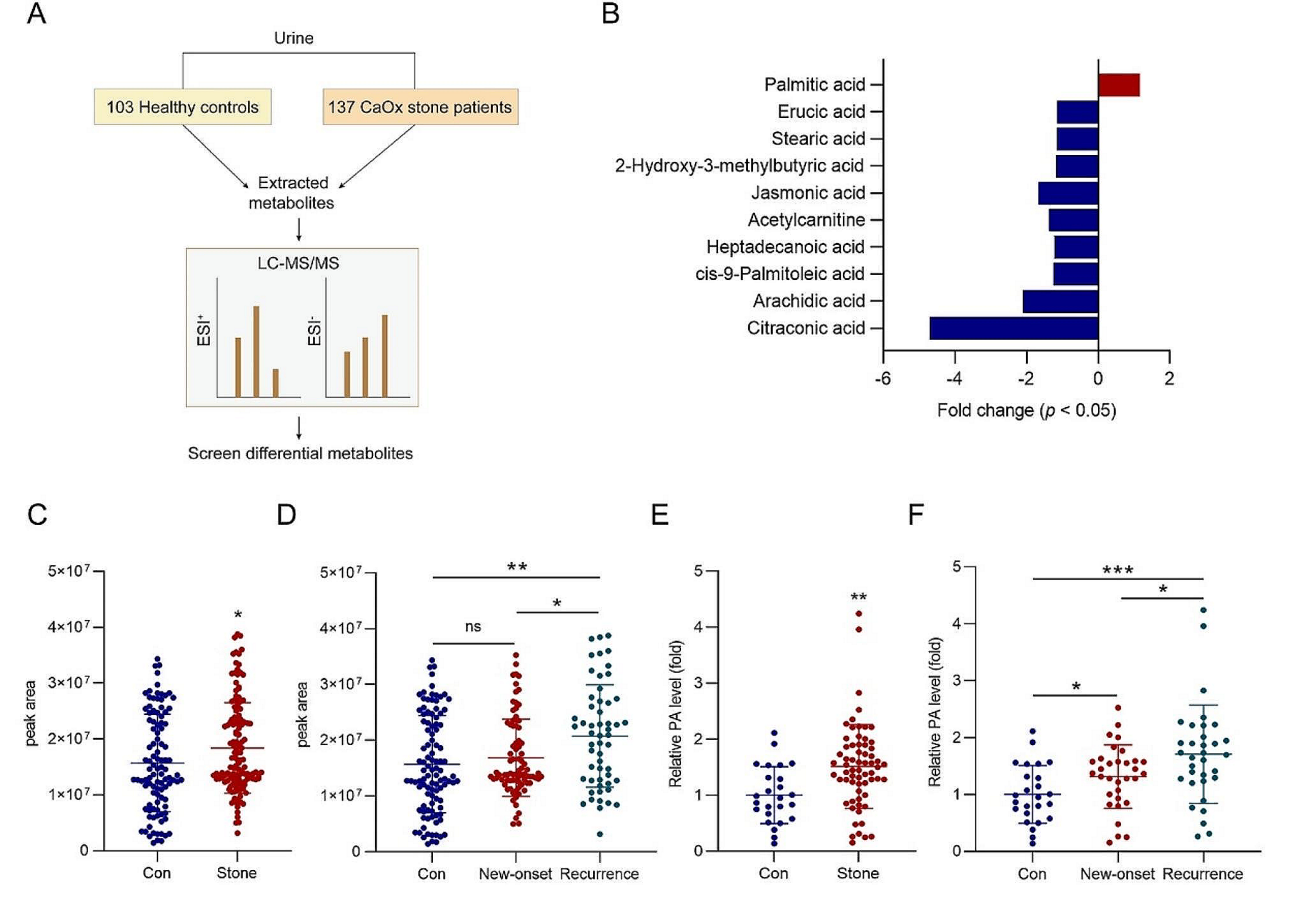
PA was increased in the urine and serum of patients with renal CaOx stones. **A** Schema of untargeted metabolomics was performed using the urine of 137 patients and 103 healthy controls. **B** PA was the only upregulated fatty acid with statistical significance (*p* < 0.05). **C** The concentration of PA in the urine of patients and the controls. **D** The comparison of PA levels in the urine between the new-onset and recurrent patients, and the controls. **E** The concentration of PA in the serum of the renal CaOx stone patients and the controls. **F** The comparison of PA level in the serum between new-onset and recurrent patients, and the controls. ^*^p < 0.05, ^**^p < 0.01, ^***^p < 0.001; ns not statistically significant

In our previous study, via metabolomics profiling and statistical analysis involving 137 patients with renal CaOx stones compared with 103 healthy controls (Fig. 1A), we identified PA as an upregulated metabolite in these patients [15]. Notably, PA was the sole upregulated fatty acyl compound with statistical significance (*p* < 0.05) (Fig. 1B) (Table S5). The concentration of PA was elevated in the urine of these patients (Fig. 1C). Although there was no significant difference in urine PA level between the new-onset patients and the controls (Fig. 1D), the relapse group exhibited higher PA levels than both the control and new-onset groups (Fig. 1D).

Furthermore, serum PA levels were also elevated in the patients compared with the control levels (Fig. 1E), with the highest levels observed in the relapse group (Fig. 1F). Interestingly, unlike the urine PA levels, serum PA levels were higher in the new-onset patients than in the controls (Fig. 1F). The identification of PA as a marker associated with the incidence and recurrence of renal CaOx stones underscores the need for further in-depth investigation.

### PA induced injury to renal tubular epithelial cells

**Fig. 2:**
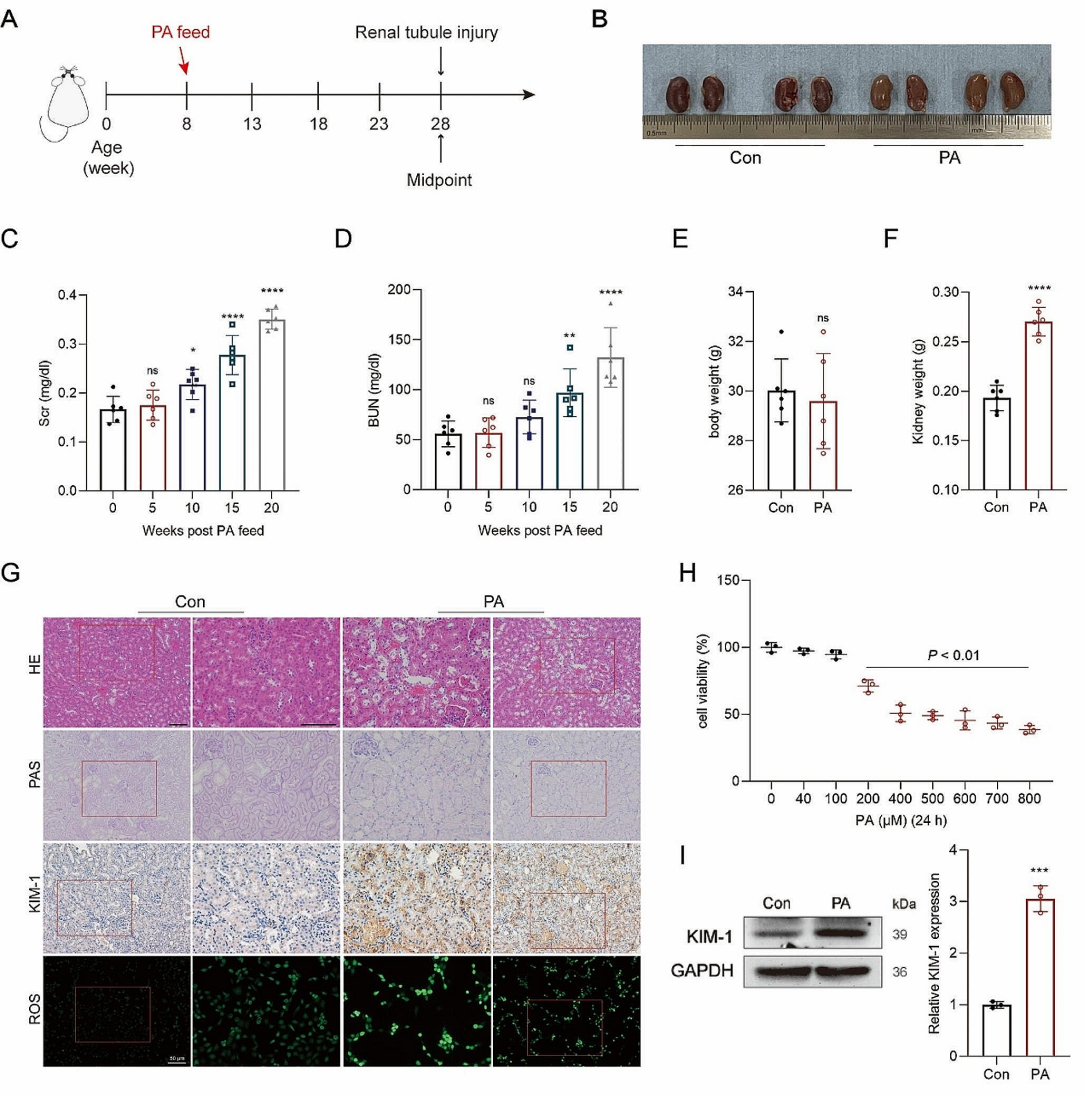
Injury of renal tubular epithelial cells was induced by PA. **A** Timeline of the mice feeding with PA and monitoring of renal tubular damage. **B** The appearance of mice kidneys from the PA group. **C-D** Changes in Scr and BUN of mice were measured dynamically. **E-F** Differences in body weight and kidney weight of the two groups. **G** HE and PAS staining were performed to observe the structural damage to renal tubular epithelial cells of the PA-treated mice, and KIM-1 level was detected by IHC; DCFH-DA probe was used to measure the levels of ROS in HK-2 cells, original magnification, 100×, scale bar, 50 μm. **H** Changes of cellular activity with gradient concentrations of PA. **I** The KIM-1 in HK-2 cells was detected by western blotting. ^*^p < 0.05, ^**^p < 0.01, ^****^p < 0.0001, ns not statistically significant, compared with the control. Scale bar for IHC, 100 μm

Mice were fed a diet containing 7.5% PA for 20 weeks (Fig. 2A), during which no significant difference in mortality was observed among the two groups. However, kidneys from the PA group exhibited a paler and slightly edematous appearance (Fig. 2B). Additionally, the Scr and BUN levels in the PA group showed a gradual increase (Fig. 2C and D). Although there was no notable difference in body weight between the two groups (Fig. 2E), the kidney weights of the mice fed with PA were increased after 20 weeks (Fig. 2F). Notably, histological analysis revealed that the renal tubular cells of the PA-treated mice displayed severe injuries, characterized by cell swelling, marked vacuolation, cell exfoliation, and loss of the brush borders (Fig. 2G). Furthermore, kidney injury molecule 1 (KIM-1) protein, a specific biomarker for renal tubular injury, was significantly upregulated in the PA-treated mice, compared with the control levels (Fig. 2G).

The IC50 of PA in HK-2 cells was estimated at approximately 400 µM via cell activity inhibition assay (Fig. 2H). Oxidative stress and excessive production of ROS were detected in PA-treated HK-2 cells (Fig. 2G). Numerous studies have highlighted the contributory role of ROS in the progression of kidney stones [21, 22]. Similarly, the addition of PA to the cell culture significantly upregulated KIM-1 (Fig. 2I). Taken together, these findings suggest that PA injures renal tubular epithelial cells.

### More CaOx crystals adhered to renal tubular epithelial cells upon the PA-induced injury

**Fig. 3:**
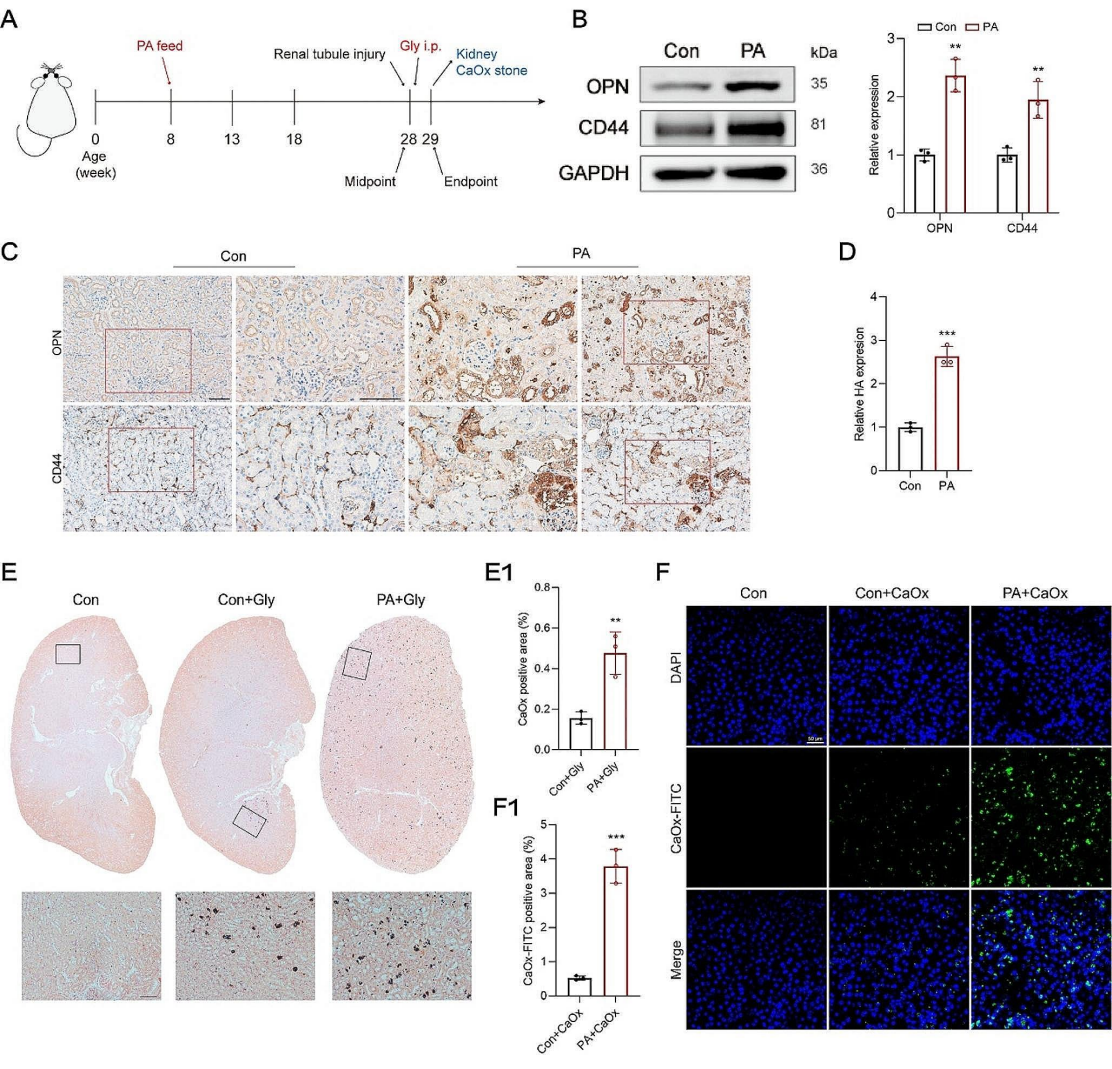
PA boosted adhesion molecule expression and promoted CaOx crystal deposition to renal tubular epithelial cells. **A** Timeline for inducing renal CaOx stone formation in the respective groups. **B-C** Western blotting and IHC were performed to measure the adhesion molecule levels of OPN and CD44 in HK-2 cells and renal tubules. **D** The level of hyaluronic acid (HA) in HK-2 cells was assayed. **E** and **E1** The deposition of CaOx crystals (dark brown) in the whole kidney was analyzed by Von Kossa staining. **F** and **F1** CaOx crystals-FITC (green) adhered to the HK-2 cell membranes were detected with fluorescence staining, original magnification, 100×, scale bar, 50 μm. ^**^p < 0.01, ^***^p < 0.001, compared with the control. Scale bar for IHC, 100 μm

Following the confirmation of injury to renal tubular epithelial cells after the 20th week, Gly (80 mg/kg/d) was intraperitoneally injected to induce the formation of renal CaOx stone in the respective groups (Fig. 3A). As the damage progressed in HK-2 cells and renal tubules within the PA group, major adhesion molecules, including CD44, osteopontin (OPN), and hyaluronic acid (HA), were upregulated (Fig. 3B-D). Interestingly, several mice from the PA group unexpectedly died on the 5th day of the Gly administration. Consequently, the model-building timeframe was adjusted to 5 days, at which point the kidneys were harvested.

More CaOx crystals were found deposited in the renal tubules of the PA-treated mice (Fig. 3E and E1). Notably, in contrast to previous studies reporting that CaOx crystals were predominantly located at the junction between the renal cortex and medulla [23, 24], we detected widespread deposition of CaOx crystals throughout the entire kidneys in the PA group (Fig. 3E).

Similarly, in HK-2 cells, more FITC-labeled CaOx crystals adhered to the cell membranes following PA treatment compared with the control level (Fig. 3F and F1). These results indicate that PA can promote renal deposition of CaOx crystals by injuring renal tubular epithelial cells.

### PA activated peroxisome proliferator-activated receptor α (PPARα), which in turn upregulated FA desaturase 1 and 2 (FADS1/2) to enhance PUFA synthesis

**Fig. 4:**
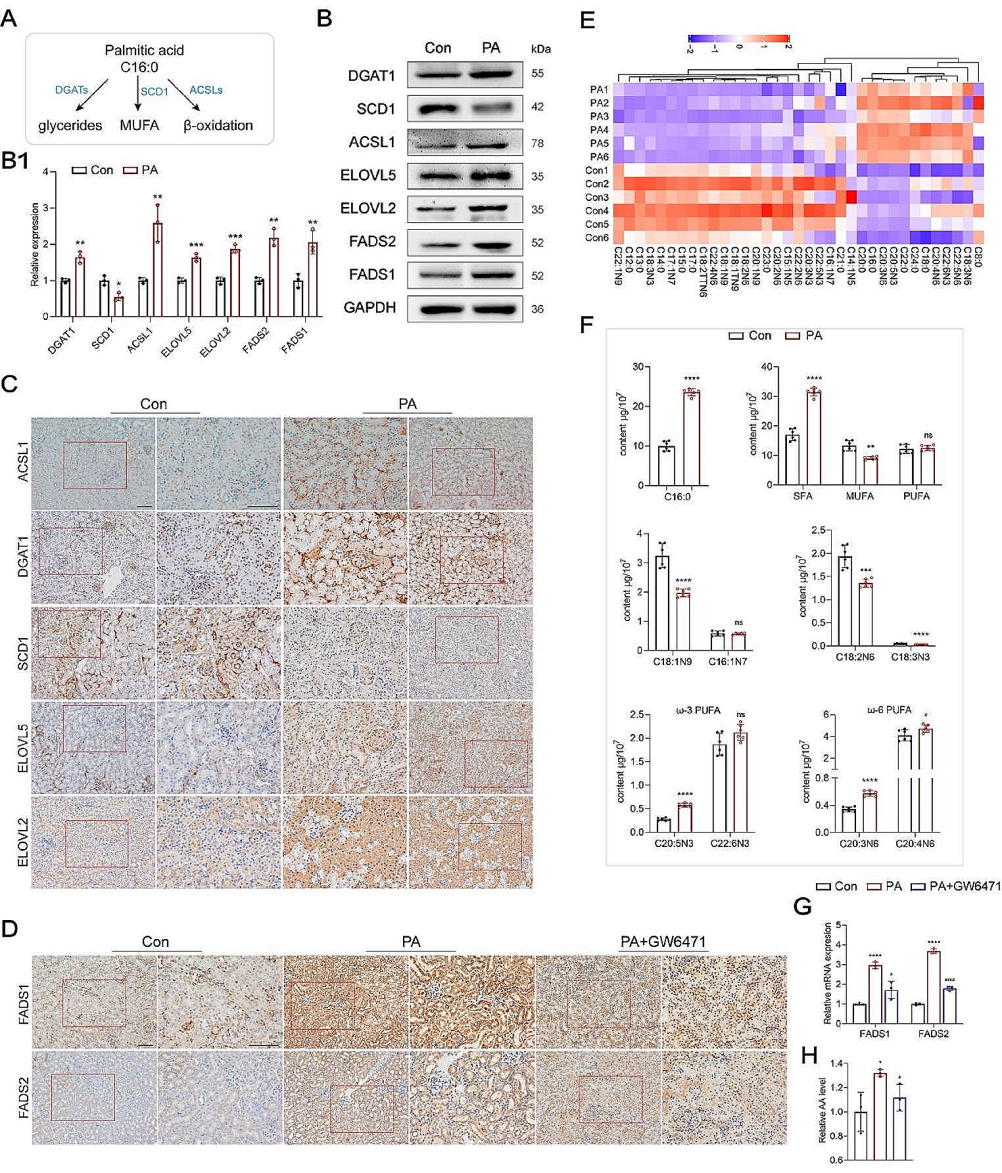
PPARα activated by PA enhanced FADS1/2 expression to upregulate PUFA synthesis. **A** Overview of the metabolic transformations of PA. **B and B1** The levels of enzymes associated with PA metabolism were evaluated by western blotting in HK-2 cells. **C** The expression of enzymes associated with PA metabolism in renal tubules was detected by IHC. **D** The FADS1/2 levels were measured in renal tubules. **E-F** Heatmap and the levels of most M/LCFAs in the PA-treated HK-2 cells via lipidomics. **G** The mRNA expression of FADS1/2 in HK-2 cells was determined by qPCR. **H** The concentration of AA in HK-2 cells was detected by the Elisa kit. ^*^p < 0.05, ^**^p < 0.01, ^***^p < 0.001, ^****^p < 0.0001, ns not statistically significant, compared with the control; ^#^*p* < 0.05, ^####^*p* < 0.0001, compared with the PA group. Scale bar for IHC, 100 μm

Acting as a central player in intracellular FA metabolism, PA undergoes several significant metabolic transformations, including glyceride synthesis, desaturation, and β-oxidation (Fig. 4A). Following the PA treatment, a comprehensive shift was observed in the levels of enzymes associated with PA metabolism in both HK-2 cells and mice kidneys. Specifically, long-chain acyl-CoA synthetase 1 (ACSL1), the initiating enzyme in β-oxidation, was activated, and diglycerol acyltransferase 1 (DGAT1) was upregulated. Conversely, the activity of stearoyl-coenzyme A desaturase 1 (SCD1), a principal Δ9 desaturase involved in MUFA desaturation, was suppressed (Fig. 4B, B1 and C). In contrast, Δ5 and Δ6 desaturases, namely FADS1 and FADS2, which are pivotal in the synthesis of ω-3 and ω-6 PUFAs, were upregulated (Fig. 4B, B1 and D). Additionally, two enzymes responsible for very long-chain FA elongation, ELOVL2 and ELOVL5, were also activated (Fig. 4B, B1 and C). These changes in PA metabolism predominantly correlated with increased β-oxidation, glyceride synthesis, and PUFA metabolism, as well as diminished MUFA desaturation.

Untargeted lipidomics analysis was employed to detect alterations in M/LCFA levels following PA treatment. The relative standard deviation (RSD) values of the quality-control samples were consistently < 30%, indicating the high precision of the measurements (Fig. S1 A and B). The levels of most M/LCFAs in the PA-treated HK-2 cells were disrupted (Fig. 4E), and the cellular PA (C16:0) concentration was increased by 2.36 folds (Fig. 4F).

Following the PA treatment, the levels of total SFAs in HK-2 cells increased by 1.85 folds (Fig. 4F). However, the major MUFAs oleic acid (C18:1N9) and palmitoleic acid (C16:1N7) were downregulated due to the suppression of SCD1 (Fig. 4F). Notably, major downstream PUFAs, including arachidonic acid (AA, C20:4N6), eicosapentaenoic acid (EPA, C20:5N3), were upregulated, whereas essential α-linoleic acid (LA, C18:2N6) and α-linolenic acid (ALA, C18:3N3) were downregulated (Fig. 4F).

It is well-established that PA cannot be directly converted into PUFAs in humans. PUFAs are synthesized through the desaturation and elongation of the essential PUFAs, catalyzed by enzymes such as FADS1 and FADS2, which utilize precursors such as LA and ALA. Intriguingly, the levels of LA and ALA decreased following the PA treatment (Fig. 4F). This observation led to the hypothesis that PA metabolism may influence the expression of FADS1 and FADS2, subsequently promoting the synthesis of the downstream PUFAs derived from LA or ALA.

Our results from protein-protein-interaction (PPI) analysis suggested that FADS1/2 is regulated by PPARα (Fig. S1C), which is a critical regulatory factor in FFA metabolism [25], abundantly expressed in proximal tubular cells. Dietary FFAs, including PA, serve as natural ligands that activate PPARα [26]. We found that the FADS1 and FADS2 levels in the PA-treated HK-2 cells and mice kidneys that were pre-treated with the selective PPARα antagonist GW6471 were lower than those not pre-treated with GW6471 (Fig. 4D and G). Consequently, the levels of AA, the downstream PUFA, also decreased (Fig. 4H).

### PA enhanced PUFA peroxidation, which in turn induced ferroptosis of renal tubular epithelial cells

**Fig. 5:**
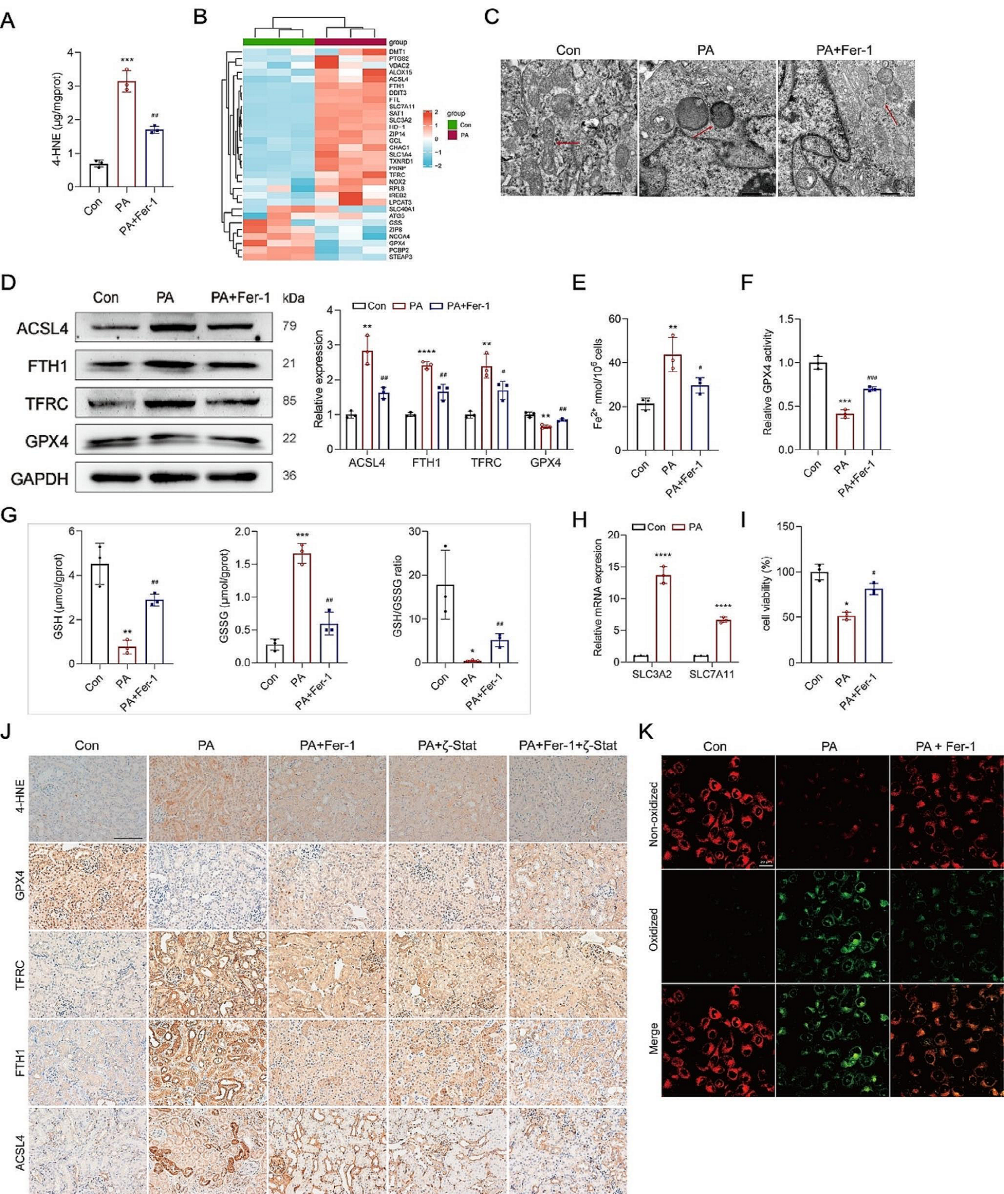
PA enhanced PUFA peroxidation to induce ferroptosis of renal tubular epithelial cells. **A** The level of lipid peroxide 4-HNE was upregulated in PA-treated HK-2 cells. **B** Heatmaps of the mRNA levels of genes related to the ferroptosis pathway were analyzed through transcriptomics. **C** changes in mitochondrial microstructure were observed through transmission electron microscopy, scale bar, 500 nm. **D** Western blotting was used to measure the protein levels of ACSL4, FTH1, TFRC, and GPX4 in HK-2 cells. **E-G** The concentration of Fe^2+^, GPX4 activity, and level of GSH, were detected in HK-2 cells. **H** The mRNA levels of SLC3A2 and SLC7A11 were detected in PA-treated HK-2 cells. **I** Cell activity was measured in PA-treated HK-2 cells. **J** The levels of 4-HNE, GPX4, TFRC, FTH1, and ACSL4 in renal tubules were evaluated by IHC. **K** Lipid peroxides in HK-2 cells were measured by BODIPY 581/591 C11 probe, original magnification, 250×, scale bar, 20 μm. ^*^p < 0.05, ^**^p < 0.01, ^***^p < 0.001, ^****^p < 0.0001, compared with the control; ^#^*p* < 0.05, ^##^*p* < 0.01, ^###^*p* < 0.001, compared with the PA group. Scale bar for IHC, 100 μm

PUFAs are susceptible to oxidation, forming lipid peroxides via the Fenton Reaction when exposed to oxidative stress. This process leads to the increased production of a specific product, 4-HNE (Fig. 5A). It is well-established that lipid peroxidation of PUFAs is a prominent feature of ferroptosis. Transcriptomics analysis revealed notable alterations in the mRNA levels of many factors associated with the ferroptosis pathway (Fig. 5B). In corroboration, the mitochondria exhibited signs of atrophy and rounding, characterized by the disappearance of cristae and increased membrane density, as observed through transmission electron microscopy (Fig. 5C). Importantly, pretreatment with Fer-1 (2 µM) (Fig. S2A), a selective and efficient inhibitor of ferroptosis, mitigated these changes (Fig. 5A and C).

The expression levels of GPX4, transferrin receptor (TFRC), ferritin heavy chain 1 (FTH1), and acyl-CoA synthetase long-chain family member 4 (ACSL4), all of which are involved in the regulation of lipid or iron metabolism [27], were changed in the PA-treated renal tubules and HK-2 cells, and these changes could be ameliorated by Fer-1 pretreatment (Fig. 5D and J). Moreover, an overload of Fe^2+^ within the PA-treated HK-2 cells was observed (Fig. 5E), accompanied by a reduction in GPX4 activity (Fig. 5F) and GSH level (Fig. 5G), despite a significant upregulation of solute carrier family 3 member 2 (SLC3A2) and solute carrier family 7 member 11 (SLC7A11) (Fig. 5H) (Fig. S2D). These observations suggest that the system xc- is not constrained by PA. Fer-1 pre-treatment mitigated the reduction in GPX4 activity and GSH level (Fig. 5F and G), and the increase in Fe^2+^ level (Fig. 5E). In parallel, the cell viability was recovered (Fig. 5I). Notably, Fer-1 pre-treatment of HK-2 cells downregulated the PUFA peroxides in the membrane of PA-treated HK-2 cells, assessed using the Bodipy C11 fluorescence dye, lipid ROS generation was inhibited (Fig. 5K).

To evaluate the impact of ferroptosis on the expression of adhesion molecules, HK-2 cells were treated with 10 µM Erastin (Fig. S2B), a specific inducer of ferroptosis. The treated cells showed significant upregulation of OPN, CD44, and TFRC (Fig. S2C).

These findings underscore the involvement of the ferroptosis pathway in renal tubular injury and the expression of adhesion molecules.

### Phosphatidic acid derived from PA activated protein kinase C (PKC) ζ, which in turn promoted the formation of the PEBP1/15-LO complex to accelerate PUFA peroxidation

In the process of ferroptosis, lipid peroxidation is not solely catalyzed by Fe^2+^ but is also influenced by lipoxygenases (LOs) [28]. The formation of the PEBP1/15-LO complex, which significantly promotes the generation of PUFA peroxides, was observed [29]. However, it is important to note that PEBP1 is bound to RAF1 under physiological conditions and dissociates from RAF1 upon phosphorylation [29]. Our results from PPI analysis suggested that PEBP1 undergoes phosphorylation through interaction with PKC ζ (Fig. S3A) [30].

**Fig. 6:**
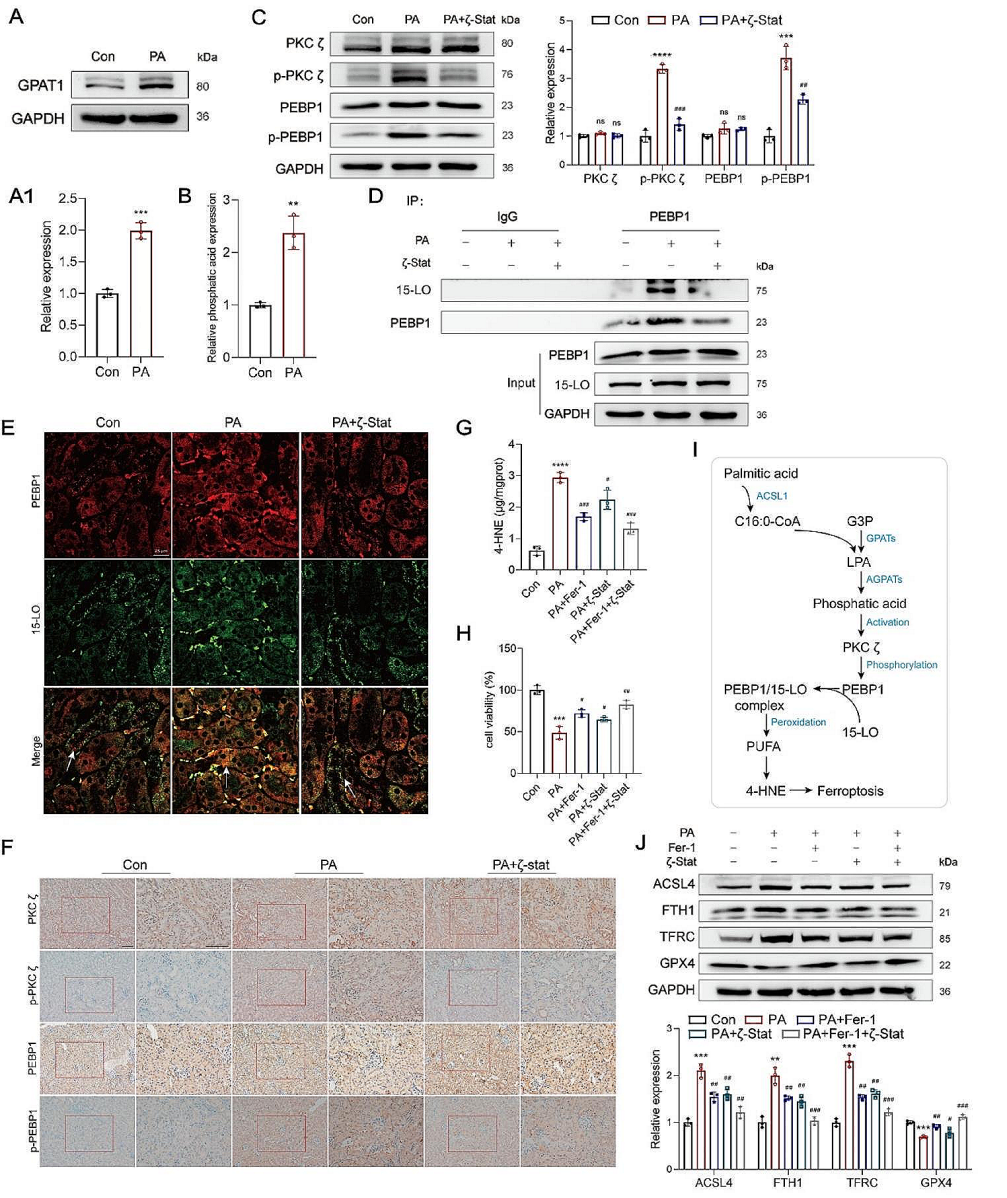
Phosphatidic acid increased by PA treatment activated PKC ζ to promote the formation of the PEBP1/15-LO complex, and accelerate PUFA peroxidation. **A** The GPAT1 level in PA-treated HK-2 cells was evaluated by western blotting. **B** The concentration of phosphatidic acid in PA-treated HK-2 cells. **C** Western blotting was performed to analyze the protein levels of PKC ζ, p-PKC ζ, PEBP1, and p-PEBP1. **D** PEBP1/15-LO complex in PA-treated HK-2 cells was detected in the CoIP experiment. **E** Immunofluorescence was used to confirm the formation of PEBP1 /15-LO complex in renal tubules, original magnification, 200×, scale bar, 25 μm. **F** The levels of PKC ζ, p-PKC ζ, PEBP1, and p-PEBP1 were measured by IHC in renal tubules. **G** The concentration of 4-HNE in PA-treated HK-2 cells after pre-treatment of Fer-1 or ζ-Stat. **H** Cell viability after Fer-1 or ζ-stat pretreatment in HK-2 cells was detected by MTT assay. **I** Hypothesis for the PUFA lipid peroxidation induced by PA promoting ferroptosis of renal tubular cells. **J** The levels of GPX4, TFRC, FTH1, and ACSL4 in HK-2 cells after pre-treatment of Fer-1 or ζ-Stat. ^**^p < 0.01, ^***^p < 0.001, ^****^p < 0.0001, compared with the control; ^#^*p* < 0.05, ^##^*p* < 0.01, ^###^*p* < 0.001, compared with the PA group. Scale bar for IHC, 100 μm

As an atypical subtype of protein kinase C, PKC ζ can be specifically activated by a limited number of second messengers, such as phosphatidic acid [31]. Interestingly, C16:0-CoA and glycerol-3-phosphate can be converted into lysophosphatidic acid (LPA) by glycerol-3-phosphate acetyltransferase 1 (GPAT1) (Fig. 6A and A1) (Fig S3C), and LPA can subsequently generate phosphatidic acid in HK-2 cells (Fig. 6B) [32].

The increased phosphorylation of PKC ζ and PEBP1 correlated with elevated levels of phosphatidic acid in both HK-2 cells and renal tubules following PA treatment (Fig. 6C). Pre-treatment with 3 µM ζ-Stat (Fig S3B), a specific inhibitor of PKC ζ, reduced the PEBP1 phosphorylation by restraining the PKC ζ phosphorylation (Fig. 6C and F). Co-immunoprecipitation experiments showed that PEBP1 was disassociated from 15-LO upon ζ-Stat pre-treatment (Fig. 6D). Furthermore, PEBP1 and 15-LO proteins co-localized more in the renal tubules of PA-fed mice than in the controls, and this effect was suppressed by the ζ-Stat pretreatment (Fig. 6E). As the ζ-Stat pre-treatment inhibited the formation of the PEBP1/15-LO complex, PUFA peroxidation decreased, resulting in downregulation of 4-HNE (Fig. 6G). Therefore, it can be inferred that PKC ζ, activated by phosphatidic acid derived from PA, phosphorylates PEBP1 to promote PUFA peroxidation catalyzed by the PEBP1/15-LO complex (Fig. 6I). With the Fer-1 or ζ-Stat pre-treatment, the increase in the 4-HNE level was suppressed, cell viability was restored (Fig. 6H), and the levels of TFRC, FTH1, GPX4, and ACSL4 were recovered in both PA-treated HK-2 cells and the kidneys of PA-treated mice (Figs. 5J and 6J) (Fig S4).

In summary, these findings underscore that PA induces the formation of the PEBP1/15-LO complex, which in turn stimulates PUFA peroxidation and ferroptosis.

### Inhibition of PUFA peroxidation reduced renal deposition of CaOx crystals

**Fig. 7:**
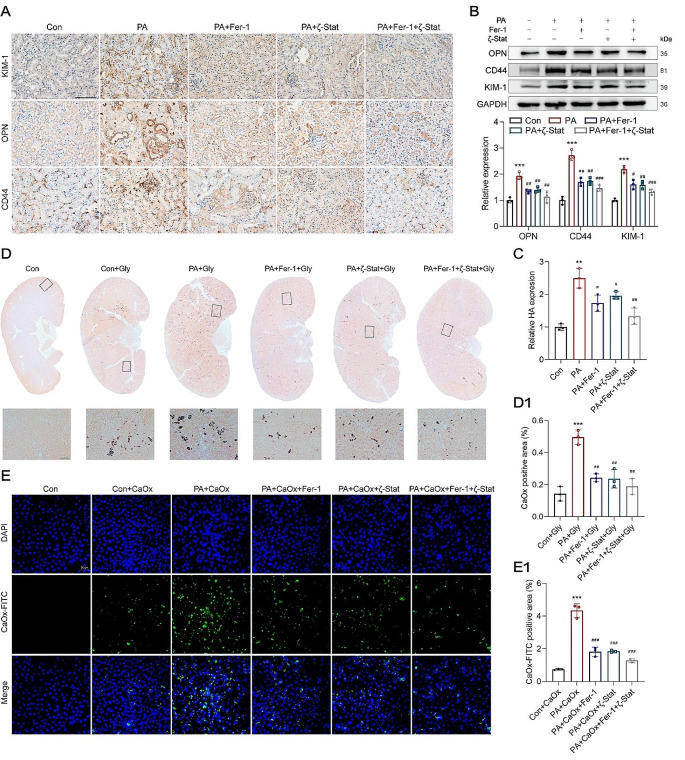
Inhibition of PUFA peroxidation reduced renal CaOx crystal deposition. **A-C** KIM-1, OPN, CD44 proteins, and HA in HK-2 cells and the kidneys of mice treated with PA were measured after pre-treatment of Fer-1 or ζ-Stat. **D** and **D1** Deposition of CaOx crystals (dark brown) in the whole kidneys was evaluated by Von Kossa staining. **E** and **E1** Fluorescence staining was performed to observe CaOx crystals-FITC (green) adhered to the HK-2 cell membranes, original magnification, 100×, scale bar, 50 μm. ^**^p < 0.01 compared with the control; ^#^*p* < 0.05, ^##^*p* < 0.01, compared with the PA group. Scale bar for IHC, 100 μm

The above findings showed that PA contributes to the formation of renal CaOx stones. As the PA-induced lipid peroxidation was alleviated by the Fer-1 or ζ-Stat pre-treatments, the PA-induced injury to renal tubular epithelial cells was alleviated and the viability of PA-treated HK-2 cells was restored (Fig. 6H). Furthermore, these pre-treatments suppressed the upregulation of KIM-1, OPN, CD44 proteins, and HA in HK-2 cells and the kidneys of mice treated with PA (Fig. 7A-C) (Fig S5). Importantly, the renal deposition of CaOx crystals was reduced (Fig. 7D and D1). Consistent with the in vivo results, the adhesion of CaOx crystals to the cell membrane was significantly suppressed in PA-treated HK-2 cells pre-treated with Fer-1 or ζ-Stat, compared with those that were not pre-treated (Fig. 7E and E1).

## Discussion

Plasma FFAs constitute a vital energy reservoir and contribute to the intermediary factors in lipid metabolism. Notably, FFAs are recognized for their capacity to induce injury to renal tubular epithelial cells, primarily through their lipotoxic effects [11, 13, 33–35]. Via nontargeted metabonomics, PA has been found to be the only upregulated FA among all the FFAs between patients with renal CaOx stones and healthy controls.

Previous studies have reported that excessive PA can increase myocardial FFA uptake and lead to mitochondrial structural remodeling with a significant reduction in minimum diameter, accompanied by loss of the mitochondrial reticulum and increased mitochondrial fission [36]. PA has also been implicated in liver fibrosis and insulin resistance. It has been shown to interfere with nucleocytoplasmic transport and glucose-induced insulin secretion [37, 38]. However, the role of PA in the formation of renal CaOx stones remains unclear.

Injury to renal tubular epithelial cells is considered to be an important cause of the formation of renal stones [39]. Changes in cell-membrane structure and adhesion sites for stone crystals happened in damaged cells [40, 41]. The adhesion molecules, such as OPN, HA, sialoglycoprotein, fibronectin, and collagen, were increased in the injured cells [42]. In the study, the expression of adhesion molecules in renal tubular epithelial cells was increased after PA treatment. In addition, CaOx crystals were more susceptible to adhering to the membrane of damaged epithelial cells.

As the hub of intracellular FFA metabolism, PA metabolism encompasses three primary pathways, including β-oxidation, glyceride synthesis, and desaturation into MUFAs [43, 44]. Notably, the expression levels of genes implicated in these three pathways were significantly changed in PA-treated renal tubular epithelial cells. Consequently, there was an overall upregulation of SFAs, whereas the principal MUFAs, namely oleic acid and palmitoleic acid, were downregulated, as revealed by our non-targeted lipidomics analysis. Although the total PUFA levels did not exhibit significant changes after PA treatment, enzymes associated with PUFA metabolism were significantly upregulated. Additionally, downstream PUFAs, such as AA, EPA, and DHA, were all found to be upregulated.

As is well known, PA cannot be directly converted into PUFAs in humans. However, LC-PUFAs can derive from LA or ALA through a consecutive series of desaturation and chain-elongation reactions mediated by FADS1/2 and ELOVLs [45, 46]. We observed that the levels of the two essential PUFAs LA and ALA were reduced in PA-treated HK-2 cells, and this change was accompanied by an increase in downstream LC-PUFA levels. Therefore, in investigating the potential association between PA and the upregulation of FADS1/2, it was established that FADS1/2 can indeed be modulated by PPARα [47, 48], which serves as the native receptor for PA [49, 50]. In corroboration, we observed that the PA-induced FADS1/2 and AA upregulation in HK-2 cells was suppressed upon their pre-treatment with the PPARα antagonist GW6471. Although PPARα inhibition by GW6471 can decrease FADSs expression, it has not been considered a therapeutic target because of its multiple regulatory functions, including promoting fatty acid oxidation and mediating inflammatory responses [51].

PUFAs are considerably vulnerable to oxidation, leading to the formation of lipid peroxides. Supplying cells with PUFAs can improve their susceptibility to ferroptosis [52]. Upon activation of ACSL4, free PUFAs can be esterified and incorporated into membrane phospholipids, a process facilitated by LPCAT3 [53]. Among the phospholipids containing PUFAs, phosphatidylethanolamines (PEs) containing AA are the predominant substrates susceptible to peroxidation during the process of ferroptosis [54].

Our transcriptomics analysis in this study revealed changes in the expression levels of genes related to the ferroptosis pathway after PA addition. Moreover, mitochondria displayed characteristic manifestations commonly associated with ferroptosis. It was found that the expression and activity of GPX4 were decreased with GSH exhaustion in the PA-treated renal tubular epithelial cells. However system xc- was not inhibited, and SLC7A11 and SLC3A2 were always upregulated. Therefore, we inferred that the reduction in GPX4 activity is mainly caused by GSH depletion. It is noteworthy that the PA-induced injury to renal tubular epithelial cells was caused by dramatic peroxidation of PUFAs, as evidenced by the results from the Fer-1 pre-treatment.

PUFA peroxidation catalyzed by LOs is another form of ferroptosis in addition to the Fenton Reaction mediated by Fe^2+^ [55]. LOs are effective in the oxygenation of free PUFAs. 15-LO can catalyze the formation of pro-ferroptosis 15-OOH-eicosatetraenoic acid (HpETE), which includes AA. HpETE is subsequently esterified into PE, forming HpETE-PE. A previous study has shown that 15-LO can form a complex with PEBP1 to accelerate PUFA peroxidation [56]. However, the role of PA in the formation of the PEBP1/15-LO complex has remained unknown. PEBP1, also known as RAF1 kinase inhibitory protein (RKIP1), is bound to and inhibits the RAF1 kinase under physiological conditions. Phosphorylation of PEBP1 disrupts its association within this complex, allowing PEBP1 to interact with new partners, including 15-LO [57, 58]. Our PPI analysis revealed that PKC ζ was involved in PEBP1 phosphorylation [30]. We found that PKC ζ can induce PEBP1 phosphorylation, thereby facilitating the binding of PEBP1 to 15-LO. PKC ζ is an atypical PKC isoform and can be activated solely by specific second messengers, such as phosphatidic acid, as opposed to conventional second messengers, including diacylglycerol (DAG) and Ca^2+^ [30, 31]. Although phosphatidic acid is one of the intermediates in the synthesis of glycerides from PA, intracellular phosphatidic acid was upregulated in HK-2 cells upon PA treatment. This upregulation occurred through a consecutive series of catalytic reactions involving ACSL1 and GPAT1. Increased formation of the PEBP1/15-LO complex due to the activation of PKC ζ increased PUFA peroxidation, which in turn induced the ferroptosis of renal tubular epithelial cells.

**Fig. 8:**
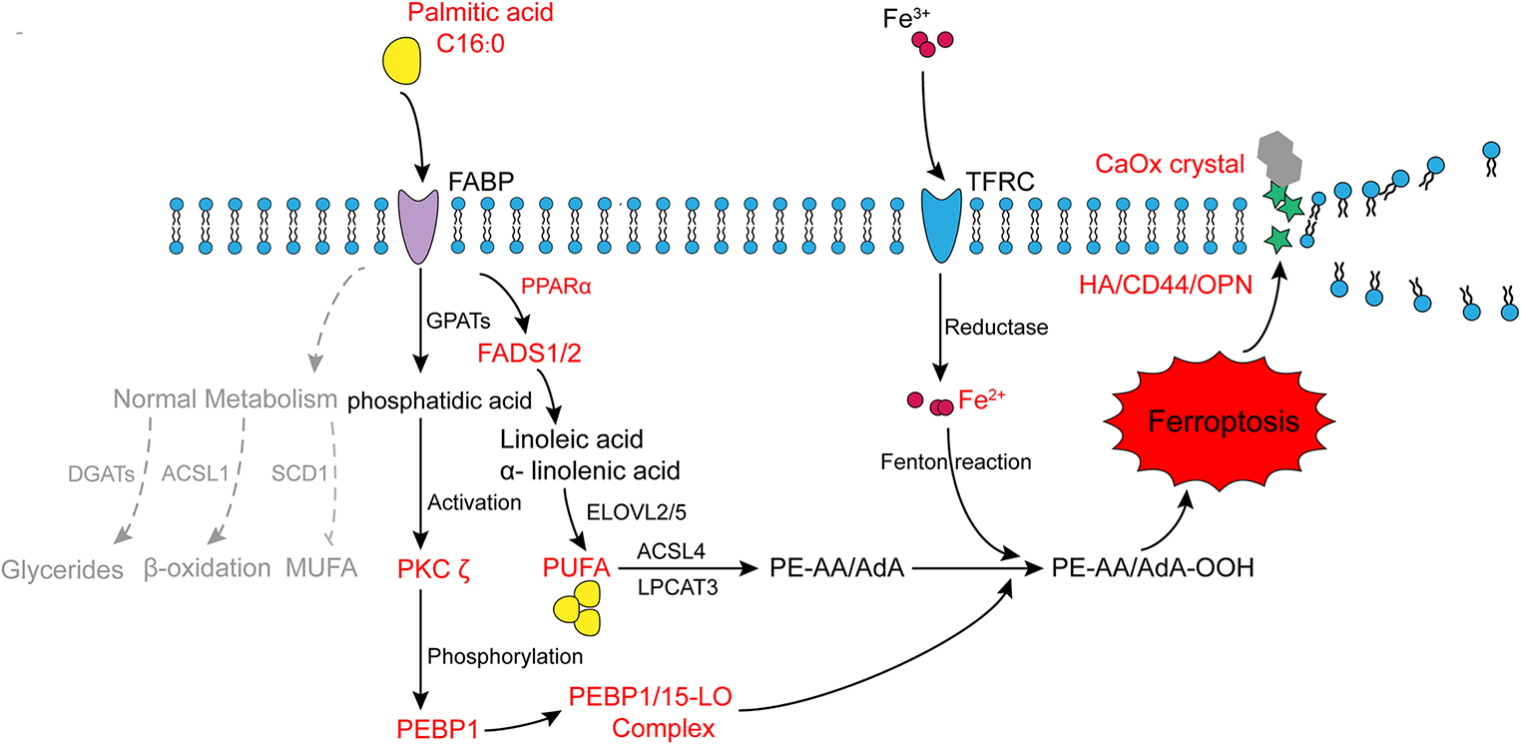
The graphical summary highlights the pathological role of PA in the formation of renal CaOx stones

Through the aforementioned experiments, we substantiated that PA, which is an SFA, upregulates PUFAs in renal tubular epithelial cells by activating PPARα. Additionally, PA promotes PUFA peroxidation by facilitating the interaction between PEBP1 and 15-LO via the activation of PKC ζ. Consequently, these processes induce ferroptosis in renal tubular cells and elevate the deposition of CaOx crystals on renal tubules due to the upregulation of adhesion molecules (Fig. 8).

In summary, our study shows that PA contributes to the formation of renal CaOx stones. Functioning as a pivotal instigator of ferroptosis by inducing downstream PUFA synthesis and peroxidation. PA causes injury to renal tubular epithelial cells and facilitates the formation of renal CaOx stones. These findings underscore the critical and intricate role of PA in nephrolithiasis.

### Electronic supplementary material

Below is the link to the electronic supplementary material.

Supplementary Material 1

## Data Availability

All data are available from the corresponding author upon reasonable request.

## Abbreviations

PA: Palmitic acid
CaOx: Calcium oxalate
MS: Metabolic syndrome
FFA: Free fatty acid
SFA: Saturated fatty acid
PAS: Periodic acid-schiff
Scr: Serum creatinine
AA: Arachidonic acid
M/LCFA: Medium- and long-chain fatty acid
Gly: Glyoxylic acid
LA: α-linoleic acid
ALA: a-linolenic acid
Fer-1: Ferrostatin-1
