# Supplementary material for: Dysregulated palmitic acid metabolism promotes the formation of renal calcium-oxalate stones through ferroptosis induced by polyunsaturated fatty acids/phosphatidic acid
Source: Cell Mol Life Sci. 2024 Feb 12;81(1):85. doi: 10.1007/s00018-024-05145-y (PMC10861707; doi:10.1007/s00018-024-05145-y)
Supplement: Supplementary file 1 — Supplementary Material 1 [file 18_2024_5145_MOESM1_ESM.pdf]

Dysregulated palmitic acid metabolism promotes the formation of renal calcium-oxalate stones through ferroptosis induced by polyunsaturated fatty acids/phosphatidic acid

Journal name: Cellular and Molecular Life Sciences

Rui Wang[a b1], Jingdong Zhang[a1], Haotian Ren[a], Shiyong Qi[a], Linguo Xie[a], Haijie Xie[a], Zhiqun Shang[a*], Chunyu Liu[a*]

[a]Department of Urology, Tianjin Institute of Urology, The Second Hospital of Tianjin Medical University, Tianjin, China.

[b]Department of Physiology and Pathophysiology, Tianjin Medical University, Tianjin, China.

[1] These authors contributed equally to this article.

[*]Corresponding author

Zhiqun Shang, Department of Urology, Tianjin Institute of Urology, The Second Hospital of Tianjin Medical University. E-mail: zhiqun_shang@tmu.edu.cn.

Chunyu Liu, Department of Urology, Tianjin Institute of Urology, The Second Hospital of Tianjin Medical University. E-mail: prof_liucy@163.com.

Supplemental methods and materials

Table S1. Clinical characteristics of both groups of urine samples.

Characteristic	Patients			Controls	*p*-value
	Total	New-onset	Recurrence		
Samples	137	55	82	103	
Age	53.12 ± 12.20	52.63±12.87	53.44±11.17	51.83 ± 14.25	0.998*
Gender[a],	95/42	40/15	55/27	61/42	0.211
Scr[b] (μM)	84.52 ± 21.09	87.05±18.57	82.82±22.57	82.42 ± 24.70	0.095*
BMI[c] (kg/m^2)	26.45 ± 3.77	25.75±3.72	26.50±3.51	25.50 ± 3.20	0.241*

*one-way ANOVA or non-parametric test (Kruskal-Wallis); [a]male/female; [b]Serum creatinine;

[c]Body mass index.

Table S2. Clinical characteristics of both groups of serum samples.

Characteristic	Patients			Controls	*p*-value
	Total	New-onset	Recurrence		
Samples	65	33	32	20	
Age	55.63 ± 10.44	55.59±10.68	55.67±10.22	53.94 ± 11.05	0.653*
Gender[a],	40/25	18/14	22/11	16/4	0.212
Cre[b] (μM)	82.36 ± 18.33	80.39±20.97	84.28±15.43	82.40 ± 21.78	0.719*
BMI[c] (kg/m^2)	26.77 ± 3.58	26.90±3.56	26.68±3.65	25.96 ± 2.42	0.610*

*one-way ANOVA; [a]male/female; [b]Serum creatinine; [c]Body mass index.

Table S3. All the antibodies were used in the study.

Antibody	Brand and Cat. NO.	Dilution in IHC	Dilution in IF	Dilution in WB
ELOVL2	Abcam 176327	1:200		1:6000
ELOVL5	Abcam 205535	1:200		1:2000
ACSL1	Abcam 177958	1:200		1:6000
FADS1	Abcam 126706	1:200		1:6000
FADS2	SAB 27350	1:200		1:2000
FTH1	Abcam 183781	1:200		1:2000
GPX4	Affinity DF6701	1:150		1:1500
ACSL4	Affinity DF12141	1:150		1:1500
4-HNE	Abcam 46545	1:100		
TFRC	Abcam 214039	1:200		1:2000
SCD1	Abcam 236868	1:200		1:2000
DGAT1	Abcam 181180	1:200		1:6000
CD44	Abcam 189524	1:100		1:2000
KIM-1	Bioss 2713R	1:150		1:1000
OPN	Huabio 0806-6	1:150		1:1500
SLC7A11	Abcam 175186	1:200		1:2000
SLC3A2	Affinity DF7468	1:150		1:1500
PKCζ	Santacruz 17781	1:100		1:8000
p-PKCζ	Santacruz 271962	1:100		1:1000
PEBP1	Santacruz 101504	1:100	1:100	1:1000

p-PEBP1	Santacruz 135779	1:100		1:1000
15-LO(mouse)	Santacruz 133085	1:100		1:1000
15-LO(rabbit)	SAB 37409	1:200	1:100	
GPAT1	Abcam 69990	1:200		1:6000

Table S4. The primer sequences were used for qPCR.

Gene		Sequences
GAPDH	Forward	ATCATCCCTGCCTCTACTGG
	Reverse	GTCAGGTCCACCACTGACAC
FADS1	Forward	GGCCCAGGAAGGCTTTCA
	Reverse	CCAGCCTTGCTGCCTCTCT
FADS2	Forward	GCACCCTTTAAGTGGCCAAT
	Reverse	TTGGCAATGGCTGGATTCCT
SLC3A2	Forward	ACTCTTCTCCTATATCCGCCACT
	Reverse	CCCACATCCCCAAAGTTAAGCAC
SLC7A11	Forward	TCTCCAAAGGAGGTTACCTGC
	Reverse	AGACTCCCCTCAGTAAAGTGAC

Table S5. Information of differential fatty acyls.

Name	Fold change	P value
Citraconic acid	0.21	1.27E-34
Arachidic acid	0.48	2.57E-11
cis-9-Palmitoleic acid	0.80	2.33E-06
Heptadecanoic acid	0.82	1.65E-03
Acetylcarnitine	0.73	1.76E-03
Jasmonic acid	0.60	2.08E-03
2-Hydroxy-3-methylbutyric acid	0.85	2.04E-02
Stearic acid	0.86	2.33E-02
Palmitic acid	1.16	2.35E-02
Erucic acid	0.88	4.76E-02

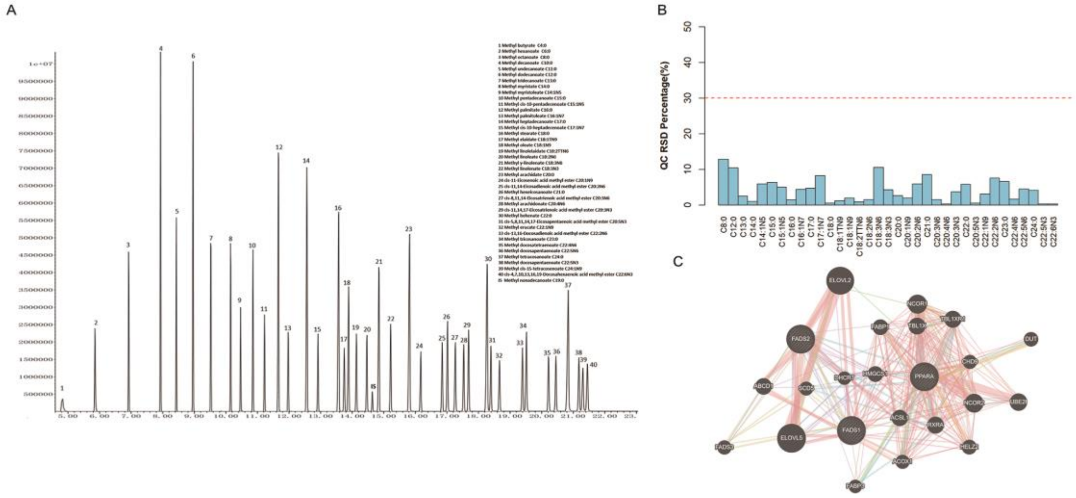

Fig. S1 PPARα activated by PA enhances FADS1/2 expression to upregulate downstream PUFA synthesis.

A The information on the peaks from each fatty acid in lipidomics was shown. **B** The relative standard deviation (RSD) of all fatty acyls in QC samples was less than 30%. **C** Protein interaction analysis of PPARα and FADS1/2 was performed.

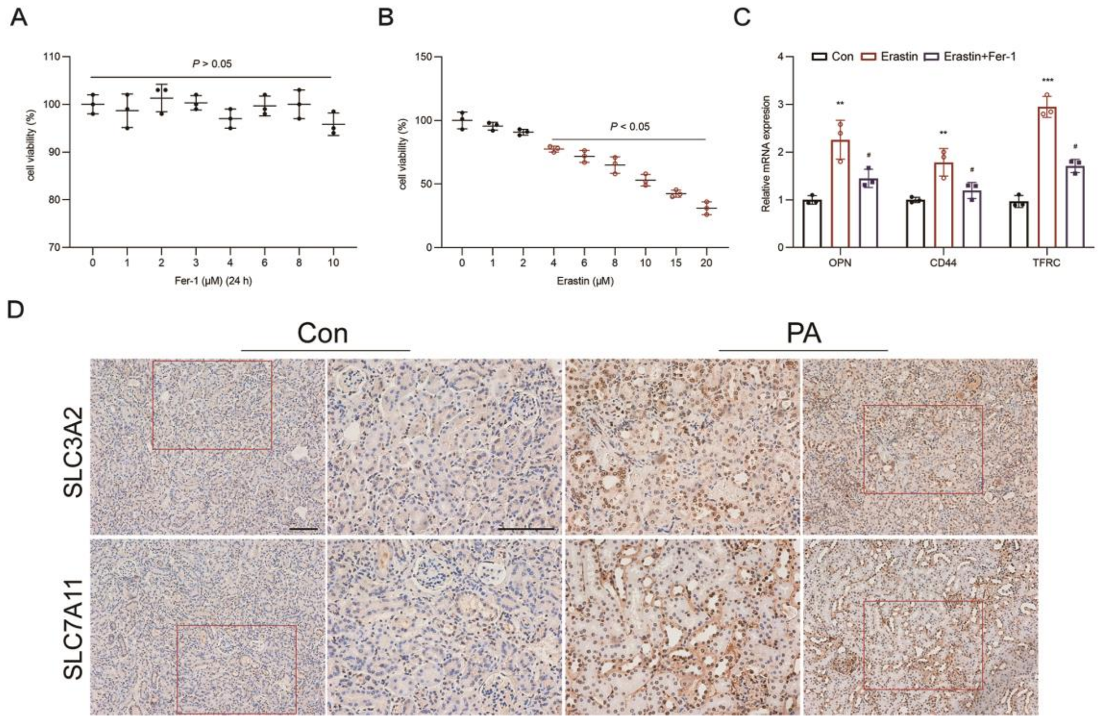

Fig. S2 PA enhanced PUFA peroxidation to induce ferroptosis of renal tubular epithelial cells.

A MTT assay was performed to screen Fer-1 optimal effect concentration. **B** MTT assay was used to screen erastin effective concentration. **C** The mRNA levels of OPN, CD44, and TFRC in HK-2 cells treated with Erastin were measured by qPCR assay. **D** The protein levels of SLC3A2 and SLC7A11 in renal tubules treated with PA were detected by IHC. **p<0.01, ***p<0.001, compared with the control; #p<0.05, compared with the PA group. Scale bar, 100 μm.

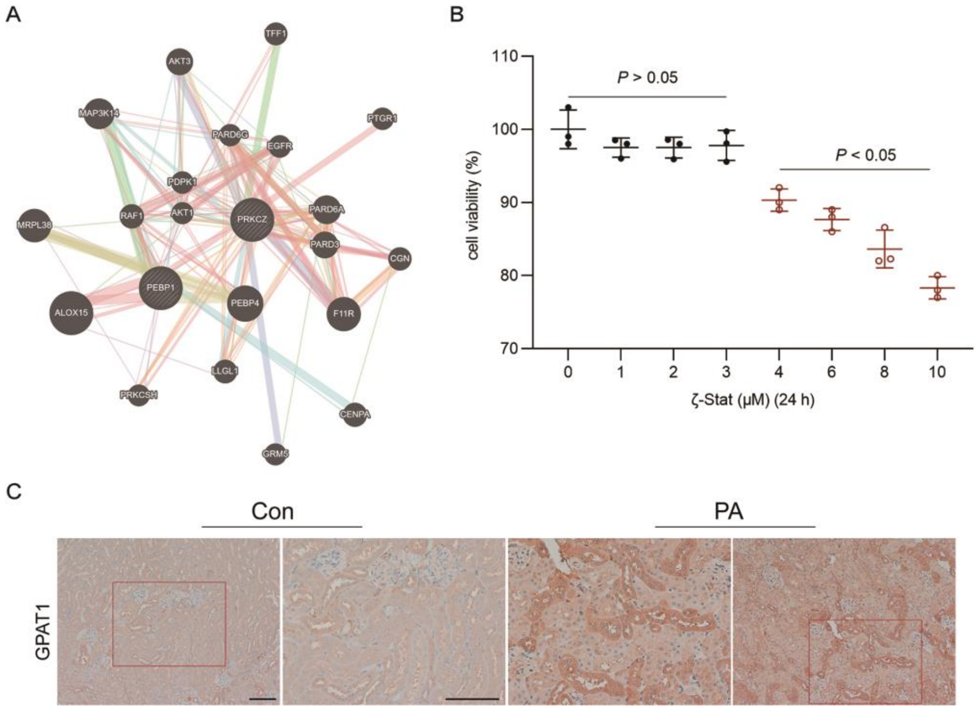

Fig. S3 Phosphatidic acid derived from PA activated PKC ζ to promote the formation of the PEBP1/15-LO complex, and accelerate PUFA peroxidation.

A Protein interaction of PKC ζ and PEBP1 was analyzed. **B** MTT assay was performed to screen ζ-Stat optimal effect concentration. **C** The expression of SLC3A2 and SLC7A11 was detected by IHC in renal tubules. Scale bar, 100 μm.

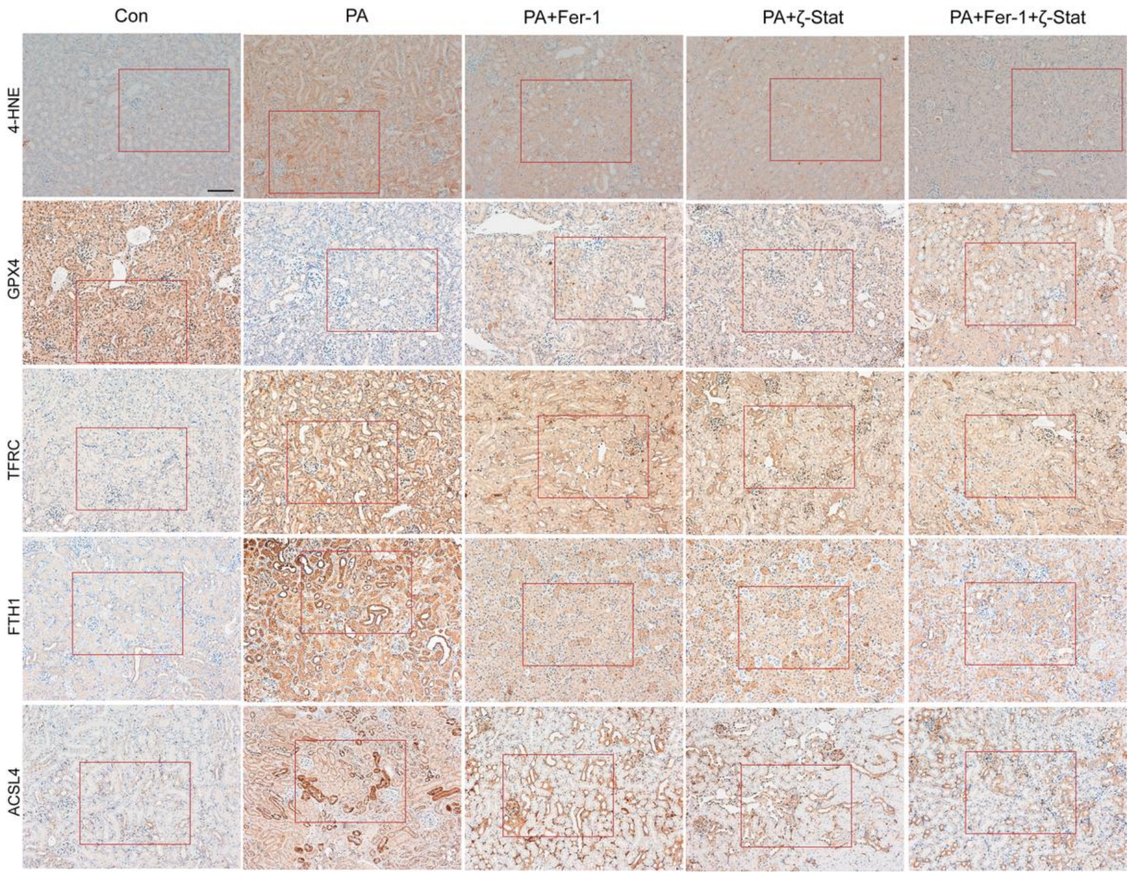

Fig. S4 PKC ζ promoted PEBP1 and 15-LO binding to accelerate the peroxidation of PUFAs.

The protein levels of 4-HNE, GPX4, TFRC, FTH1, and ACSL4 in renal tubules were evaluated by IHC (100×). Scale bar, 100 μm.

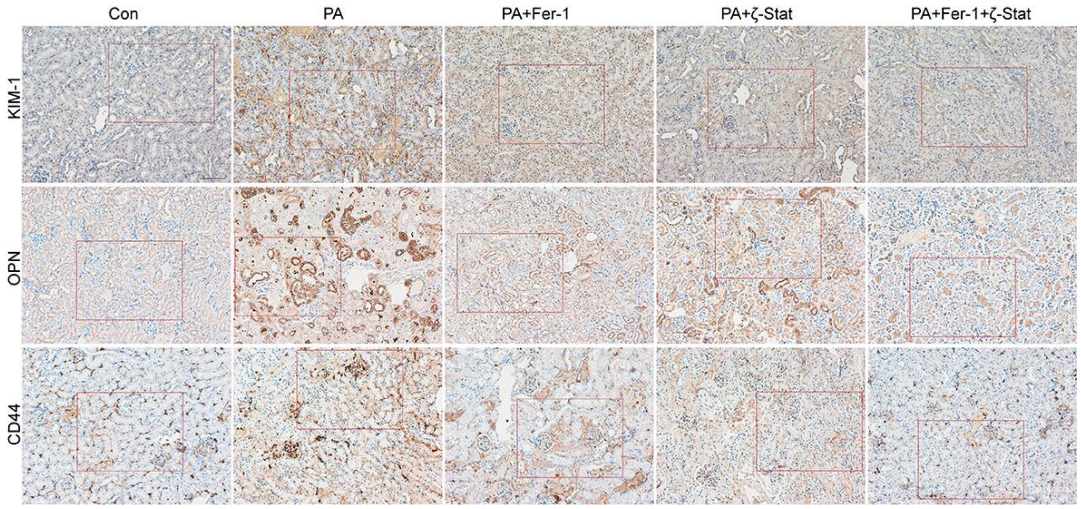

Fig. S5 Inhibition of PUFA peroxidation reduced renal CaOx crystal deposition.

The protein levels of KIM-1, OPN, and CD44 in renal tubules were evaluated by IHC (100×). Scale bar, 100 μm.

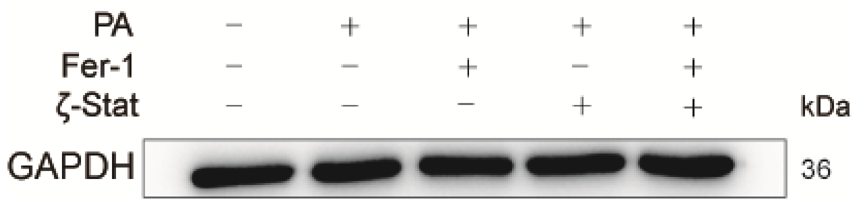

Fig. S6 . The internal control levels for OPN in Fig 7.

The protein levels of GAPDH in the five groups.
